# Pain is associated with reduced adherence to safer injection practices among people who inject opioids

**DOI:** 10.1186/s12954-026-01490-2

**Published:** 2026-06-19

**Authors:** O. Trent Hall, John A. Sturgeon, Jasmin Bradley, Abigail Helm, Johnathan Rausch, Emma Kuloszewski, Megan Deaner, Carlos Malvestutto, Ashley Lipps, Afton Hassett

**Affiliations:** 1 Department of Psychiatry and Behavioral Health, Ohio State University Wexner Medical Center Talbot Hall, 181 Taylor Ave., Columbus, OH 43203 USA; 2 Department of Anesthesiology, University of Michigan Medical School, Ann Arbor, MI USA; 3 Safe Point Harm Reduction Program, Equitas Health, Columbus, OH USA; 4 Department of Medicine, Division of Health Systems Science, University of Massachusetts Chan Medical School, Worcester, MA USA; 5 Ohio University Heritage College of Osteopathic Medicine Dublin Campus, Columbus, OH USA; 6 Division of Infectious Diseases Columbus, Ohio State University College of Medicine, Columbus, OH USA

**Keywords:** Harm reduction, Needle-exchange programs, Risk behaviors, Safer injection practices, Opioid-related disorders, Pain

## Abstract

**Background:**

Injection opioid use is associated with fatal and non-fatal harms including accidental overdose and infectious diseases. Syringe service programs offer sterile injection equipment and safer injection training to reduce the incidence of injection-related harms. Nevertheless, factors that might reduce adherence to safer injection practices are understudied. This report explores pain as one such factor.

**Methods:**

People who inject opioids (n = 130) were recruited from a syringe service program for a cross-sectional survey assessing the relationship between fluctuations in pain severity and adherence to safer injection practices over the past 7 days.

**Results:**

A within-subjects Sign Test analysis revealed participants’ adherence to safer injection practices fluctuated with changes in pain severity. When their pain was at its worst, participants reported they less often engaged in hand washing (Z −5.63,* P* < .001), cleaning the injection site (Z −5.29, * P*  < .001), and waiting until in a clean safe place to inject (Z −3.94, * P* < .001). Additionally, they more often reused their own injection equipment (Z −2.55, P = .011), and injected faster, or at a higher dose (Z −2.71,* P* = .007) when in worse pain.

**Conclusions:**

This study provides initial evidence that pain is associated with reduced adherence to safer injection practices among people who inject opioids. Although all study participants received training in safer injection practices and cost-free sterile injection equipment, within-subject adherence to safer use practices varied significantly under conditions of best and worst pain.

## Introduction

Injection opioid use—defined as subcutaneous (SQ), intramuscular (IM), or intravenous (IV) injection of opioids such as fentanyl or heroin—is associated with a variety of adverse health outcomes [17, 18, 33]. It has been estimated that 14.8 million people are engaged in injection drug use globally [18]. People who inject drugs, including people who inject opioids, are at risk of accidental overdose and life-threatening infections [17]. A recent meta-analysis estimated 3.2 million people who inject drugs had experienced at least one overdose in the previous year [10]. In the United States, nearly 80,000 people died of fatal drug overdose in 2024 and opioids were involved in nearly 70% of these deaths [23]. Recent analyses suggest that while opioid-smoking-related deaths now outnumber injection-related opioid overdoses, tens of thousands of overdose deaths are injection-related each year [26, 49].

Infectious complications of injection drug use may include soft tissue infections, sepsis, endocarditis, osteomyelitis, spinal epidural abscess, human immunodeficiency virus / acquired immunodeficiency syndrome (HIV/AIDS), and viral hepatitis (hepatitis B and hepatitis C viruses) (HBV and HCV) [33, 34]. Fifteen percent of people who inject are believed to be living with HIV and more than 38% may have a current HCV infection [18]. Because of the prevalence and severity of these adverse outcomes, reducing harms associated with injection drug use is a growing clinical, scientific, and public health priority.

Harm reduction is defined by the National Harm Reduction Coalition as *“a set of practical strategies and ideas aimed at reducing negative consequences associated with drug use* (“Harm Reduction Principles,” n.d.)*.”* Syringe service programs offer harm reduction interventions for people who inject opioids. Such interventions most often include provision of sterile injection equipment and education regarding *‘safer use practices’* intended to reduce the risk of accidental overdose and prevent the transmission of communicable disease [38, 50]. Research into the effectiveness of syringe service programs has been stymied by political, legal, social, and financial barriers to their implementation and sustainability [31]. Indeed, such constraints have been posited to limit the availability and effectiveness of these programs at reducing drug-related harms [40, 45]. Nevertheless, harm reduction services have been shown to prevent transmission of infectious diseases and accidental overdose among people who inject drugs, and research in this area has become a funding priority of the National Institute on Drug Abuse (NIDA) and Substance Abuse and Mental Health Service Administration (SAMHSA) (Abuse, 2022; [7],*Harm *[27, 41, 44]).

The extent to which pain might interfere with adherence to safer injection practices for people who inject opioids is unknown. Pain is defined by the International Association for the Study of Pain (IASP) as “an unpleasant sensory and emotional experience associated with, or resembling that associated with, actual or potential tissue damage [35].” This definition explicitly acknowledges the complexity of pain as multidimensional construct inclusive of a wide range of experiential domains and causal etiologies. Chronic pain is prevalent among individuals with opioid use disorder (OUD) and a recent study of syringe service program clients found that a majority of people who inject opioids perceived pain to be related to the onset, maintenance, and relapse of their OUD [42, 48]. Prior research has demonstrated an association between pain and non-medical opioid use among people who inject opioids [12]. However, to our knowledge, no study has explored whether increased pain intensity might influence the behavior of people who inject opioids such that they forgo learned safer injection practices to expedite pain relief.

Pain-related negative urgency is believed to play an important role in the relationship between chronic pain and substance use [21]. Negative urgency refers to the tendency to act impulsively when distressed [21]. Given that negative urgency has been linked to a variety of harmful or risky behaviors, it is plausible that pain might motivate people who inject opioids to eschew learned safer injection practices [11, 16, 25]. If true, pain might be a previously unrecognized factor limiting engagement in and adherence to safer injection practices. By extension, linking people who inject opioids to evidence-based pain management might reduce their risk of accidental overdose and infectious complications of injection drug use.

Therefore, this study aimed to determine if fluctuations in pain severity over the past 7 days were associated with changes in injection practices among people who inject opioids who had received standardized safer injection training. Specifically, we assessed whether adherence to safer use practices differed when pain was at its best and worst severity over the preceding 7 days. We hypothesized that participants would report lower adherence to safer injection practices during periods of worst pain compared to best pain.

## Materials and methods

### Setting

This research was conducted at Equitas Health through their Safe Point program—a syringe service program in Columbus, Ohio—between July 25, 2023, and March 19, 2024. Safe Point provides comprehensive harm reduction services intended to prevent accidental overdose and transmission of infectious diseases. These services include syringe exchange, infectious disease screening, medical and behavioral health linkage, case management, and education regarding safer injection practices. Standardized safer injection education is provided in verbal and written form to all program clients by trained syringe service program staff. This education includes advising clients about specific behaviors to reduce their risk of infection and overdose (i.e., clean hands and skin prior to injection, avoid using alone, etc.).

### Participants

Adult syringe service program clients (n = 191) who had previously received standardized safer use education were consecutively recruited for a larger study about substance use. Exclusion criteria for the larger study were inability to provide informed consent, read, or understand survey items. The present sub-analysis includes participants who endorsed injecting opioids (IV, IM, or SQ) 2 or more times per day and rated their average pain intensity 3 or more out of 10 over the past 7 days (n = 130). A pain threshold of 3/10 was selected to ensure adequate sample size while focusing on individuals experiencing pain that might reasonably influence injection behaviors.

### Procedures

Recruitment was conducted by a trained Research Coordinator while syringe exchange program clients waited for harm reduction services. To reduce risk of bias, participants completed questionnaires in private and were not allowed to interact with one another while completing the survey. Survey data were collected in English using REDCap, a web platform designed for secure collection of personal health information and online database management [29].

Participants completed a screening battery assessing bodily pain and headache the Chronic Overlapping Pain Condition Screener (COPCS) [46]. They were then asked to rate the severity of their best, worst, and average pain over the past 7 days on a numerical rating scale from 0 (no pain) to 10 (worst pain imaginable) [20]. Participants then responded to a series of questions assessing their injection opioid use practices when their pain was at its best and worst severity. These questions were based on safer injection practices counseling and scored 1 = never, 2 = rarely, 3 = sometimes, 4 = often, 5 = always. Table 1 displays relevant questionnaire items.

**Table 1:** Questions regarding pain severity and injection practices during best pain and worst pain

1. In the past 7 days, how would you rate your average pain?
2. In the past 7 days, how would you rate your pain at its worst?
3. In the past 7 days, how would you rate your pain at its best?
4. When your pain is at its _*, as you rated above, how often do you…
a. Clean your hands before injecting drugs
b. Clean your skin at the site where you plan to inject drugs
c. Reuse your own injection equipment (needles, syringes, filters, cookers, etc. you have injected with at least once before)
d. Borrow used injection equipment, or share injection equipment with someone else
e. Wait to inject drugs until you can get to a place that is clean, and you feel safe

Responses to questions 1–3 were rated on a 0 to 10 numeric scale with 0 indicating ‘no pain’ and 10 indicating ‘worst pain imaginable.’ Question 4 included sub-items a-e and was asked 3 separate times for best pain, worst pain and average pain. _* = best, worst, average. These sub-items were scored 1 = never, 2 = rarely, 3 = sometimes, 4 = often, 5 = always

### Statistical analysis

Descriptive statistics detailed sample characteristics as well as pain and substance use variables. A within-subjects analysis was conducted to determine whether participants’ adherence to safer injection practices varied with changes in pain severity (best pain and worst pain over the preceding 7 days). Paired self-report ratings of adherence to safer injection practices during best pain and worst pain were compared via Sign Test. The Sign Test is a non-parametric alternative to the paired sample t-test which is more statistically conservative than the Wilcoxon Signed-Rank Test for the type of data presented in this study. This analytical method was chosen due to its suitability for paired, ordinal data and ability to assess the direction of behavioral change (i.e., whether participants endorsed greater, or lesser adherence to safer injection practices under conditions of best and worst pain severity). Statistical significance was set at *p* < 0.05. To account for multiple comparisons across 7 outcomes, we used the Benjamini & Hochberg procedure to control the false discovery rate [5]. All analyses were performed with SPSS software Version 28.0.

### Ethics

The study protocol (2022H0369) was approved by the Ohio State University Wexner Medical Center Institutional Review Board (Located: Columbus, Ohio, initial approval granted on 11/27/2022). Participants provided verbal consent and were monetarily compensated for their time.

## Results

### Sample characteristics

Demographic information was given by 129 (99.2%) participants. The majority identified as White (n = 120, 92.3%), male (n = 78, 60.0%), and Non-Hispanic (126, 96.9%). On average, participants were 38.4 years old (SD = 8.5) and had used opioids for 17.0 years (SD = 8.0). Nearly all endorsed IV opioid use 128 (98.5%). Other injection opioid use was less common, with 13 (10%) reporting IM and 9 (6.9%) SQ opioid use. All participants included in this analysis injected (IV, IM, or SQ) opioids at least twice daily. The mean number of times participants used opioids daily was 5.4 (SD = 3.5). One hundred eleven (111, 85.4%) of participants met diagnostic criteria for at least one chronic pain condition [46]. The most common chronic pain condition in the sample was chronic low back pain (87, 66.9%). A comprehensive presentation of pain characteristics in this sample is available elsewhere [43]. Demographic and opioid use data are summarized in Table 2.

**Table 2:** Sample characteristics

Characteristic	Participants (n = 130)
*Age mean (SD)*	
Years	38.4 (8.5)
*Sex assigned at birth n (%)*	
Female	52 (40.0)
Male	78 (60.0)
*Racial identity n (%)*	
Black or African American	8 (6.2)
White or Caucasian	120 (92.3)
Native American or Alaskan Native	6 (4.6)
Native Hawaiian or Pacific Islander	1 (0.8)
Asian	1 (0.8)
*Ethnicity n (%)*	
Hispanic	3 (2.3)
Non-Hispanic	126 (96.9)
*Age of first opioid use mean (SD)*	
Years	21.3 (8.2)
*OUD severity mean (SD)*	
DSM-5 criteria	10.2 (1.6)
*Route of administration n (%)*	
Intravenous	128 (98.5)
Subcutaneous	9 (6.9)
Intramuscular	13 (10.0)
Smoking	87 (66.9)
Swallowing by mouth	17 (13.1)
Snorting / sniffing up the nose	43 (33.1)
Another Route	4 (3.1)
*Monetary cost of daily opioid use mean (SD)*	
US dollars	85.4 (59.0)

One participant was missing age, race and ethnicity data. Sixteen were missing data for monetary cost of daily opioid use

### Pain severity and adherence to safer injection practices

**Fig. 1:**
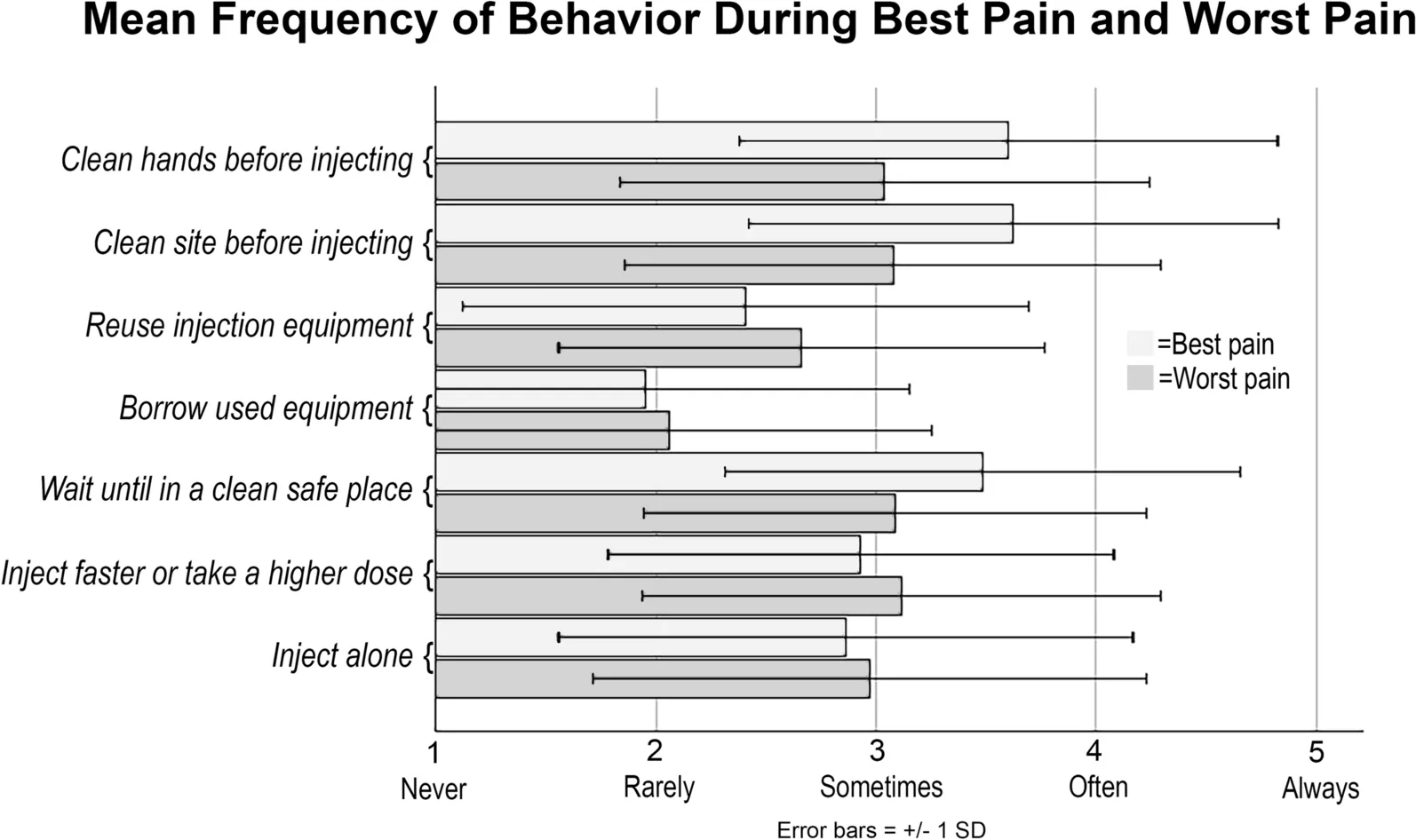
Mean self-reported frequency of injection practices under conditions of best and worst pain

Participants were asked to rate their pain at its best, worst, and average severity over the past 7 days on a 0–10 visual analog scale. Mean best pain severity was 4.8 (SD = 2.1). Mean average pain severity was 6.5 (SD = 2.1). Mean worst pain severity was 7.7 (SD = 1.9). Participants were also asked to report how often they engaged in specific injection practices when their pain was at its best and worst severity. Figure 1 displays mean self-reported frequency of injection practices under conditions of best and worst pain.

### Within subject differences in injection practices during best and worst pain

A within-subjects Sign Test analysis revealed participants’ adherence to 5 of 7 safer injection practices fluctuated with changes in pain severity (best pain versus worst pain over the preceding 7 days). When their pain was at its worst, participants less often engaged in hand washing, cleaning the injection site, and waiting until in a clean safe place to inject. Additionally, participants were significantly more likely to reuse their own injection equipment, and inject faster, or at a higher dose when their pain was at its worst. No significant difference was observed in the frequency of borrowing, or sharing others’ injection equipment, or injecting alone when pain was at its worst relative to when pain was at its best. Table 3 displays the results of the within-subject analysis.

**Table 3:** Results of sign tests comparing injection practices during best pain and worst pain

Behavior	Increase (n)	Decrease (n)	No change (n)	Z	*p*-value
Clean hands before injection	8	53	69	−5.63	< 0.001
Clean skin at injection site	10	53	67	−5.29	< 0.001
Reuse injection equipment	45	23	62	−2.55	0.011
Borrow or share injection equipment	27	15	88	−1.70	0.090
Wait to inject until in a clean safe place	15	47	68	−3.94	< 0.001
Inject faster or use a higher dose	41	19	70	−2.71	0.007
Inject alone	29	17	84	−1.62	0.105

The Increase column contains the number of participants who increased the frequency of each behavior when their pain was at its worst severity. The Decrease column reflects the number of participants who engaged in each behavior less when their pain was at its worst severity. All significant findings remained significant after Benjamini–Hochberg correction (FDR = 0.05)

## Discussion

This study provides initial evidence that pain is associated with reduced adherence to safer injection practices among people who inject opioids. Although all study participants received training in safer injection practices and cost-free sterile injection equipment, within-subject adherence to safer use practices varied significantly under conditions of best and worst pain. When their pain was at its worst, participants reported they were more likely to engage in behaviors placing them at increased risk of infectious complications of injection opioid use and accidental overdose. These findings suggest pain may be a previously overlooked factor increasing risks associated with injection opioid use and a potential target for behavioral interventions to improve adherence to safer injection counseling among syringe service program clients.

The public health burden associated with injection drug use has been described as a ‘crisis’ [34, 51]. The prevalence of injection drug use in the United States is likely underestimated, but 1,000,000 Americans may engage in injection drug use annually [6, 34]. These individuals are at increased risk of accidental overdose, as well as life-threatening infectious complications including viral, bacterial, and fungal infections of the skin, soft tissue, osteoarticular joint spaces, eyes, blood, liver, and heart [36]. The Infectious Disease Society of America (IDSA) and the HIV Medicine Association have called for expansion of harm reduction programs and services to address this crisis [34]. Convergent evidence demonstrates syringe service programs reduce transmission of infectious disease and prevent overdose [4, 22, 39]. However, little is known about factors that might increase the risk of poor outcomes among syringe service program clients or limit adherence to the safer injection practices they teach.

Prior research linking pain, impulsivity, and risk taking provides context to the present findings. Pain is a cognitively demanding experience and therefore may impair other cognitive processes like decision making [47]. Participants in the present study reported decreased adherence to safer injection practices when their pain was more severe, possibly reflecting impaired decision making under the increased cognitive load imposed by worsening pain. Indeed, experimentally induced acute pain increases other impulsive risk-taking behavior among healthy adults [32]. Aligning with our observations, individuals with chronic pain exhibit disrupted risk decision-making when compared to healthy adults [30]. Additionally, impulsivity, especially negative urgency, or the tendency to act impulsively when distressed, has been associated with opioid misuse and symptoms of OUD among people with chronic pain [21, 52]. Pain has also been shown to negatively impact quality of life among syringe service clients [15]. Together, this prior research provides a potential explanation of why many participants in the present study eschewed learned safer injection practices when their pain intensity increased. However, to our knowledge, no prior study has examined how pain might impair risk decision-making among people with OUD, or people who inject drugs. Our report contributes to the literature by showing that pain severity might affect risk decision making among people who inject opioids, possibly leading to more frequent engagement in behaviors associated with life threatening infections and accidental overdose.

The present study had notable limitations. Due to practical and ethical considerations, injection opioid use could not be directly observed. Self-report measures are susceptible to memory errors. Consistent with existing self-report pain surveys, we attempted to minimize memory errors by limiting the recall window to 7 days [3, 8]. Additionally, this study used a cross-sectional design, eliminating the opportunity for determining causal or temporal relationships between pain and injection opioid use. Specific domains of impulsivity, including negative urgency, were not directly assessed. Nevertheless, a growing body of literature substantiates the role of negative urgency in the relationship between pain and substance use as well as other risky behaviors [16, 21, 24, 37, 52]. Although participants completed a validated questionnaire screening for a variety of chronic pain conditions (COPCS), the etiology of pain was not assessed by clinician evaluation or by medical record review. Among people with OUD, reports of pain may relate to disease, injury, symptoms of opioid hyperalgesia, opioid withdrawal and related negative affective urgency, or a combination of etiologies. However, according to the IASP’s definition of pain, “a person’s report of an experience of pain should be respected” regardless of etiology, or where in the body or nervous system it might arise [35]. This assertion is especially salient in harm reduction research and clinical practice. Nevertheless, more thorough assessment of specific pain etiologies would have substantially strengthened our study as the present design could not determine whether specific pain mechanisms differentially contributed to reduced adherence to safer injection practices. Finally, participants were recruited from a single syringe service program and were predominantly White, non-Hispanic, and male. English-only data collection may limit generalizability.

Future research should replicate and extend the present work while accounting for its limitations. Longitudinal observational research should explore pain as one possible factor reducing adherence to safer injection practices and examine pain-related negative urgency as a potential target for behavioral intervention among syringe service program clients. The addition of medical and psychological pain treatments to syringe service programs should be tested as a possible means to reduce harms of injection opioid use. Current best practices for treatment of chronic pain includes a personalized, multimodal, interdisciplinary treatment approach consisting of a combination of medications, psychological interventions, integrative therapies, and procedures [9]. There is growing evidence for psychological interventions in the treatment of chronic pain in adults [13, 14, 19]. The efficacy and optimal delivery of these interventions for people who inject opioids is unknown. However, syringe service programs already provide linkage to a variety of health services (i.e., vaccinations, primary care, mental health services, etc.) [2]. Therefore, referral to pain treatment would align with the existing scope of these programs.

In conclusion, this study offers initial evidence that fluctuations in pain intensity may be associated with changes in adherence to safer use practices among people who inject opioids. A within-subjects analysis revealed that participants were less adherent to safer use practices when their pain was worse. To our knowledge, no prior study has shown pain to motivate higher risk injection practices among people who inject opioids. Given the tremendous public health burden associated with injection drug use, pain should be explored as a potential target for interventions to increase adherence to safer use practices among people who inject opioids.

## Data Availability

Data used in this study are available from the corresponding author upon reasonable request.

## Abbreviations

SQ: Subcutaneous
IM: Intramuscular
IV: Intravenous
HIV/AIDS: Human Immunodeficiency Virus / Acquired Immunodeficiency Syndrome
HBV: Hepatitis B virus
HCV: Hepatitis C virus
NIDA: National Institute on Drug Abuse
SAMHSA: Substance Abuse and Mental Health Service Administration
OUD: Opioid use disorder

## References

[1] Abuse, N. I. on D. (2022, October 26). *Harm Reduction | National Institute on Drug Abuse (NIDA)*. https://nida.nih.gov/research-topics/harm-reduction

[2] Adams JM. Making the case for syringe services programs. Public Health Rep. 2020;135(1_suppl):10S-12S. 32735192 10.1177/0033354920936233 PMC7407057

[3] Amtmann D, Cook KF, Jensen MP, Chen W-H, Choi S, Revicki D, et al. Development of a PROMIS item bank to measure pain interference. Pain. 2010;150(1):173–82. 20554116 10.1016/j.pain.2010.04.025 PMC2916053

[4] Aspinall EJ, Nambiar D, Goldberg DJ, Hickman M, Weir A, Van Velzen E, et al. Are needle and syringe programmes associated with a reduction in HIV transmission among people who inject drugs: a systematic review and meta-analysis. Int J Epidemiol. 2014;43(1):235–48. 24374889 10.1093/ije/dyt243

[5] Benjamini Y, Hochberg Y. Controlling the false discovery rate: a practical and powerful approach to multiple testing. J Roy Stat Soc: Ser B (Methodol). 1995;57(1):289–300.

[6] Bradley H, Rosenthal EM, Barranco MA, Udo T, Sullivan PS, Rosenberg ES. Use of population-based surveys for estimating the population size of persons who inject drugs in the United States. J Infect Dis. 2020;222(Supplement_5):S218–29. 10.1093/infdis/jiaa318. 32877538 PMC8176905

[7] Campbell EM, Jia H, Shankar A, Hanson D, Luo W, Masciotra S, et al. Detailed transmission network analysis of a large opiate-driven outbreak of HIV infection in the United States. J Infect Dis. 2017;216(9):1053–62. 29029156 10.1093/infdis/jix307 PMC5853229

[8] Cleeland CS, Ryan K. The brief pain inventory. Pain Research Group. 1991;20:143–7.

[9] Cohen SP, Vase L, Hooten WM. Chronic pain: an update on burden, best practices, and new advances. The Lancet. 2021;397(10289):2082–97. 10.1016/S0140-6736(21)00393-7. 34062143

[10] Colledge S, Peacock A, Leung J, Larney S, Grebely J, Hickman M, et al. The prevalence of non-fatal overdose among people who inject drugs: a multi-stage systematic review and meta-analysis. Int J Drug Policy. 2019;73:172–84. 31351755 10.1016/j.drugpo.2019.07.030

[11] Cyders MA, Smith GT. Emotion-based dispositions to rash action: positive and negative urgency. Psychol Bull. 2008;134(6):807. 18954158 10.1037/a0013341 PMC2705930

[12] Dahlman D, Kral AH, Wenger L, Hakansson A, Novak SP. Physical pain is common and associated with nonmedical prescription opioid use among people who inject drugs. Subst Abuse Treatm, Prevent Policy. 2017;12:1–11. 28558841 10.1186/s13011-017-0112-7 PMC5450090

[13] Darnall BD. Psychological treatment for patients with chronic pain. American Psychological Association, Washington. 2019.

[14] de C Williams, A. C., Fisher, E., Hearn, L., & Eccleston, C. Psychological therapies for the management of chronic pain (excluding headache) in adults. Cochrane Database Systemat Rev. 2020;8. 10.1002/14651858.CD007407.pub4 PMC7437545 32794606

[15] Deaner ME, Rausch J, Entrup P, Hall OT. Untreated opioid use disorder and health-related quality of life among syringe service program clients. JAMA Netw Open. 2024;7(4):e245968–e245968. 38578642 10.1001/jamanetworkopen.2024.5968 PMC10998153

[16] Deckman T, Nathan DeWall C. Negative urgency and risky sexual behaviors: a clarification of the relationship between impulsivity and risky sexual behavior. Personality Individ Differ. 2011;51(5):674–8. 10.1016/j.paid.2011.06.004.

[17] Degenhardt L, Charlson F, Stanaway J, Larney S, Alexander LT, Hickman M, et al. Estimating the burden of disease attributable to injecting drug use as a risk factor for HIV, hepatitis C, and hepatitis B: findings from the Global Burden of Disease Study 2013. Lancet Infect Dis. 2016;16(12):1385–98. 27665254 10.1016/S1473-3099(16)30325-5

[18] Degenhardt L, Webb P, Colledge-Frisby S, Ireland J, Wheeler A, Ottaviano S, et al. Epidemiology of injecting drug use, prevalence of injecting-related harm, and exposure to behavioural and environmental risks among people who inject drugs: a systematic review. Lancet Glob Health. 2023. 10.1016/s2214-109x(23)00057-8. 36996857 PMC12580156

[19] Driscoll MA, Edwards RR, Becker WC, Kaptchuk TJ, Kerns RD. Psychological Interventions for the treatment of chronic pain in adults. Psychol Sci Public Interest. 2021;22(2):52–95. 10.1177/15291006211008157. 34541967

[20] Farrar JT, Young JP Jr, LaMoreaux L, Werth JL, Poole RM. Clinical importance of changes in chronic pain intensity measured on an 11-point numerical pain rating scale. Pain. 2001;94(2):149–58. 11690728 10.1016/S0304-3959(01)00349-9

[21] Ferguson E, Zale E, Ditre J, Wesolowicz D, Stennett B, Robinson M, et al. CANUE: a theoretical model of pain as an antecedent for substance use. Ann Behav Med. 2021;55(5):489–502. 10.1093/abm/kaaa072. 32914834 PMC8122475

[22] Fernandes RM, Cary M, Duarte G, Jesus G, Alarcão J, Torre C, et al. Effectiveness of needle and syringe programmes in people who inject drugs—an overview of systematic reviews. BMC Public Health. 2017;17(1):309. 28399843 10.1186/s12889-017-4210-2 PMC5387338

[23] Garnett, M. F., & Miniño, A. M. (2026). *Drug Overdose Deaths in the United States, 2023–2024*. 10.15620/cdc/174639 PMC12969052 41770984

[24] Gilmour C, Blaes S, Bush NJ, Vitus D, Carpenter RW, Robinson M, et al. Pain and alcohol consumption in virtual reality. Exp Clin Psychopharmacol. 2023;31(2):433. 36174144 10.1037/pha0000602 PMC10033379

[25] Gonçalves SF, Izquierdo AM, Bates RA, Acharya A, Matto H, Sikdar S. Negative urgency linked to craving and substance use among adults on buprenorphine or methadone. J Behav Health Serv Res. 2024;51(1):114–22. 37414999 10.1007/s11414-023-09845-4 PMC11002981

[26] Hall EW, Sullivan PS, Bradley H. Estimated number of injection-involved overdose deaths in US states from 2000 to 2020: secondary analysis of surveillance data. JMIR Public Health Surveill. 2024;10:e49527. 38578676 10.2196/49527 PMC11031697

[27] *Harm Reduction*. (2023, February 22). https://www.samhsa.gov/find-help/harm-reduction

[28] Harm Reduction Principles. (n.d.). *National Harm Reduction Coalition*. Retrieved March 29, 2024, from https://harmreduction.org/about-us/principles-of-harm-reduction/

[29] Harris PA, Taylor R, Minor BL, Elliott V, Fernandez M, O’Neal L, et al. The REDCap consortium: building an international community of software platform partners. J Biomed Inform. 2019;95:103208. 31078660 10.1016/j.jbi.2019.103208 PMC7254481

[30] Hess, L. E., Haimovici, A., Muñoz, M. A., & Montoya, P. Beyond pain: modeling decision-making deficits in chronic pain. Frontiers in Behav Neurosci. 2014;8. 10.3389/fnbeh.2014.00263 PMC4117932 25136301

[31] Jones CM. Syringe services programs: an examination of legal, policy, and funding barriers in the midst of the evolving opioid crisis in the US. Int J Drug Policy. 2019;70:22–32. 31059965 10.1016/j.drugpo.2019.04.006

[32] Koppel L, Andersson D, Morrison I, Posadzy K, Västfjäll D, Tinghög G. The effect of acute pain on risky and intertemporal choice. Exp Econ. 2017;20(4):878–93. 10.1007/s10683-017-9515-6. 29151807 PMC5665967

[33] Larney S, Peacock A, Mathers BM, Hickman M, Degenhardt L. A systematic review of injecting-related injury and disease among people who inject drugs. Drug Alcohol Depend. 2017;171:39–49. 28013096 10.1016/j.drugalcdep.2016.11.029

[34] Levitt, A., Mermin, J., Jones, C. M., See, I., & Butler, J. C. Infectious diseases and injection drug use: public health burden and response. J Infect Dis. 2020;222(Supplement_5):S213–S217. 10.1093/infdis/jiaa432 32877539

[35] Loeser, J. (2021). Terminology | International Association for the Study of Pain. *International Association for the Study of Pain (IASP)*. https://www.iasp-pain.org/resources/terminology/

[36] Marks L, Nolan N, Liang S, Durkin M, Weimer M. Infectious complications of injection drug use. Med Clin North Am. 2021;106(1):187–200. 10.1016/j.mcna.2021.08.006. 34823730

[37] Moskal D, Loughran TA, Funderburk JS, Scharer JL, Buckheit KA, Beehler GP. Pain and hazardous alcohol use in veterans in primary care: the role of affective pain interference and alcohol pain-coping perceptions. J Pain. 2023. 10.1016/j.jpain.2023.09.020. 37783381

[38] Phillips KT, Stein MD, Anderson BJ, Corsi KF. Skin and needle hygiene intervention for injection drug users: results from a randomized, controlled Stage I pilot trial. J Subst Abuse Treat. 2012;43(3):313–21. 22341554 10.1016/j.jsat.2012.01.003 PMC3358564

[39] Platt, L., Minozzi, S., Reed, J., Vickerman, P., Hagan, H., French, C., Jordan, A., Degenhardt, L., Hope, V., & Hutchinson, S. (2017). Needle syringe programmes and opioid substitution therapy for preventing hepatitis C transmission in people who inject drugs. Cochrane Database of Systemat Rev. (9). 10.1002/14651858.CD012021.pub2 PMC5621373 28922449

[40] Pridgen BE, Bontemps AP, Lloyd AR, Wagner WP, Kay ES, Eaton EF, et al. US substance use harm reduction efforts: a review of the current state of policy, policy barriers, and recommendations. Harm Reduct J. 2025;22(1):101. 40484964 10.1186/s12954-025-01238-4 PMC12147315

[41] Puzhko S, Eisenberg MJ, Filion KB, Windle SB, Hébert-Losier A, Gore G, et al. Effectiveness of interventions for prevention of common infections among opioid users: a systematic review of systematic reviews. Front Public Health. 2022;10:749033. 35273933 10.3389/fpubh.2022.749033 PMC8901608

[42] Rausch J, Entrup P, Deaner M, King J, Hall OT. Pain and its perceived relatedness to the onset, maintenance and relapse of opioid use disorder: a descriptive study of non-treatment-seeking individuals. Canadian J Pain. 2024;8:2332198. 10.1080/24740527.2024.2332198 PMC11110686 38778924

[43] Rausch, J., Harte, S. E., Williams, D. A., Clauw, D. J., Deaner, M., Brodsky, L., Floyd, J., & Hall, O. T. Chronic overlapping pain conditions in individuals with active opioid use disorder: a descriptive study of syringe program participants. J Addict Dis. 2025;1–10. 10.1080/10550887.2025.2541133 40898582

[44] Ruiz MS, O’Rourke A, Allen ST, Holtgrave DR, Metzger D, Benitez J, et al. Using interrupted time series analysis to measure the impact of legalized syringe exchange on HIV diagnoses in Baltimore and Philadelphia. JAIDS J Acquir Immune Def Syndromes. 2019;82:S148–54. 31658203 10.1097/QAI.0000000000002176 PMC6820712

[45] Saloner B, Heller D, Davis CS, Sherman SG. Harm reduction: the neglected pillar of US drug policy. Annu Rev Public Health. 2025;46(1):369–87. 39689280 10.1146/annurev-publhealth-071723-112620

[46] Schrepf A, Maixner W, Fillingim R, Veasley C, Ohrbach R, Smith S, et al. The chronic overlapping pain condition screener. J Pain. 2023. 10.1016/j.jpain.2023.08.009. 37633574 PMC13390051

[47] Silvestrini, N., & Corradi-Dell’Acqua, C. The impact of pain on subsequent effort and cognitive performance. J Psychophysiol. 2022.

[48] Speed TJ, Parekh V, Coe W, Antoine D. Comorbid chronic pain and opioid use disorder: literature review and potential treatment innovations. Int Rev Psychiatry. 2018;30(5):136–46. 30398071 10.1080/09540261.2018.1514369

[49] Tanz LJ. Routes of drug use among drug overdose deaths—United States, 2020–2022. MMWR Morb Mortal Wkly Rep. 2024. 10.15585/mmwr.mm7306a2. 38358969 PMC10899081

[50] Thakarar K, Nenninger K, Agmas W. Harm reduction services to prevent and treat infectious diseases in people who use drugs. Infect Dis Clin North Am. 2020;34(3):605–20. 32782104 10.1016/j.idc.2020.06.013 PMC7596878

[51] Vearrier L. The value of harm reduction for injection drug use: a clinical and public health ethics analysis. Dis Mon. 2019;65(5):119–41. 10.1016/j.disamonth.2018.12.002. 30600096

[52] Vest N, Reynolds CJ, Tragesser SL. Impulsivity and risk for prescription opioid misuse in a chronic pain patient sample. Addict Behav. 2016;60:184–90. 10.1016/j.addbeh.2016.04.015. 27156219

